# Expression of CD38 in myeloma bone niche: A rational basis for the use of anti-CD38 immunotherapy to inhibit osteoclast formation

**DOI:** 10.18632/oncotarget.17896

**Published:** 2017-05-16

**Authors:** Federica Costa, Denise Toscani, Antonella Chillemi, Valeria Quarona, Marina Bolzoni, Valentina Marchica, Rosanna Vescovini, Cristina Mancini, Eugenia Martella, Nicoletta Campanini, Chiara Schifano, Sabrina Bonomini, Fabrizio Accardi, Alberto L. Horenstein, Franco Aversa, Fabio Malavasi, Nicola Giuliani

**Affiliations:** ^1^ Department of Medicine and Surgery, University of Parma, Parma, Italy; ^2^ Laboratory of Immunogenetics, Department of Medical Sciences and CeRMS, University of Torino, Torino, Italy; ^3^ Clinical Medicine Unit, Department of Medicine and Surgery, University of Parma, Parma, Italy; ^4^ Pathology, “Azienda Ospedaliero-Universitaria di Parma”, Parma, Italy; ^5^ Hematology and BMT Center, “Azienda Ospedaliero-Universitaria di Parma”, Parma, Italy; ^6^ CoreLab, “Azienda Ospedaliero-Universitaria di Parma”, Parma, Italy

**Keywords:** multiple myeloma, CD38, osteoclast, bone disease, immunotherapy

## Abstract

It is known that multiple myeloma (MM) cells express CD38 and that a recently developed human anti-CD38 monoclonal antibody Daratumumab mediates myeloma killing. However, the expression of CD38 and other functionally related ectoenzymes within the MM bone niche and the potential effects of Daratumumab on bone cells are still unknown. This study firstly defines by flow cytometry and immunohistochemistry the expression of CD38 by bone marrow cells in a cohort of patients with MM and indolent monoclonal gammopathies. Results indicate that only plasma cells expressed CD38 at high level within the bone niche. In addition, the flow cytometry analysis shows that CD38 was also expressed by monocytes and early osteoclast progenitors but not by osteoblasts and mature osteoclasts. Indeed, CD38 was lost during *in vitro* osteoclastogenesis. Consistently, we found that Daratumumab reacted with CD38 expressed on monocytes and its binding inhibited *in vitro* osteoclastogenesis and bone resorption activity from bone marrow total mononuclear cells of MM patients, targeting early osteoclast progenitors. The inhibitory effect was not observed from purified CD14^+^ cells, suggesting an indirect inhibitory effect of Daratumumab. Interestingly, *all-trans* retinoic acid treatment increased the inhibitory effect of Daratumumab on osteoclast formation.

These observations provide a rationale for the use of an anti-CD38 antibody-based approach as treatment for multiple myeloma-induced osteoclastogenesis.

## INTRODUCTION

Multiple myeloma (MM) is an incurable plasma cell (PC) malignancy leading to osteolytic bone disease, due to an increased formation and activity of osteoclasts (OCs) and a decreased osteoblastogenesis [1–3]. The relationship between MM cells and the bone cells, OCs and osteoblasts (OBs), plays a critical role both in the progression of MM and in the development of osteolytic bone lesions. Malignant PCs highly express surface molecules involved in the adhesion to the bone marrow (BM) microenvironment cells and in the development of bone lesions [4, 5].

Among the adhesion molecules, CD38 is highly expressed by MM cells [6–9] although its pathophysiological role in MM and in MM-induced bone disease is apparently more complex. CD38 is a 45-kDa type II transmembrane glycoprotein, widely expressed by several cell types [8], which plays a dual role as a receptor and ectoenzyme [10]. It is involved in T cell activation and proliferation, B cell differentiation and cell adhesion through the non-substrate ligand CD31 [11]. Moreover, CD38 acts as metabolic sensor that converts NAD+ to cADPR and ADPR and NADP+ to NAADP+, according to pH status.[12] CD38 acts in conjunction with the other ectoenzymes CD73 and CD203a, in the alternative axis of extracellular production of the immunosuppressive factor adenosine (ADO), bypassing the canonical pathway mediated by CD39 [13]. In addition to the transmembrane arrangement, a CD38 soluble form also exists, probably as a result of enzymatic cleavage of the cell-surface protein, both in normal and pathological fluids [14] and in exosomes [15]. CD38 is also involved in the remodelling of the adult skeleton in mice [16], being expressed on murine OCs [1, 17] and OBs [18]. Moreover, Sun L *et al*. showed that rabbit OCs expressed CD38 on plasma membrane, with ADP ribosyl cyclase activity, and an anti-CD38 agonist antibody inhibited bone resorption [17]. However, the expression of CD38 by human OCs and OBs has not yet been reported.

Daratumumab (DARA), a high-affinity human IgG1κ anti-CD38 monoclonal antibody (mAb), showed encouraging results in the treatment of patients with relapsed or refractory disease [19–21]. It has a broad-spectrum killing activity that includes complement-dependent cytotoxicity (CDC), antibody-dependent cell-mediated cytotoxicity (ADCC), antibody-dependent cellular phagocytosis (ADCP), apoptosis and, at least in part, modulation of CD38 enzymatic activity [22]. A study from Nijhof IS *et al*. [23] showed that DARA-induced CDC and ADCC were strictly associated to the level of CD38 and the pre-treatment with all-*trans* retinoic acid (ATRA) up-regulated CD38 expression in MM cells, enhancing DARA effects in a humanized MM mouse model [23].

Microvesicles (MVs) are a class of extracellular vesicles shed by different cell types under physiological conditions. Moreover, patients with different types of cancer were reported to have high number of circulating MVs suggesting their direct involvement in modulating cell communication within the tumor microenvironment. More importantly, the hypoxic microenvironment may accelerate the release of MVs into the surrounding environment promoting tumor growth, invasion and angiogenesis [24, 25]. Recently, Horenstein AL *et al*. [26] have shown that DARA ligation on MM cells was followed by aggregation, polarization, and release of MVs, extrusions of cell membrane that bear CD38 on the surface, whose fate needs to be defined [26].

Currently, the expression of CD38 and its related ectoenzymes by OCs and OBs in MM BM niche and the effects of DARA on MM-induced bone remodelling alterations are still unknown and were investigated in this study.

## RESULTS

### Expression profiling of CD38 in the bone niche of MM patients

**Figure 1:**
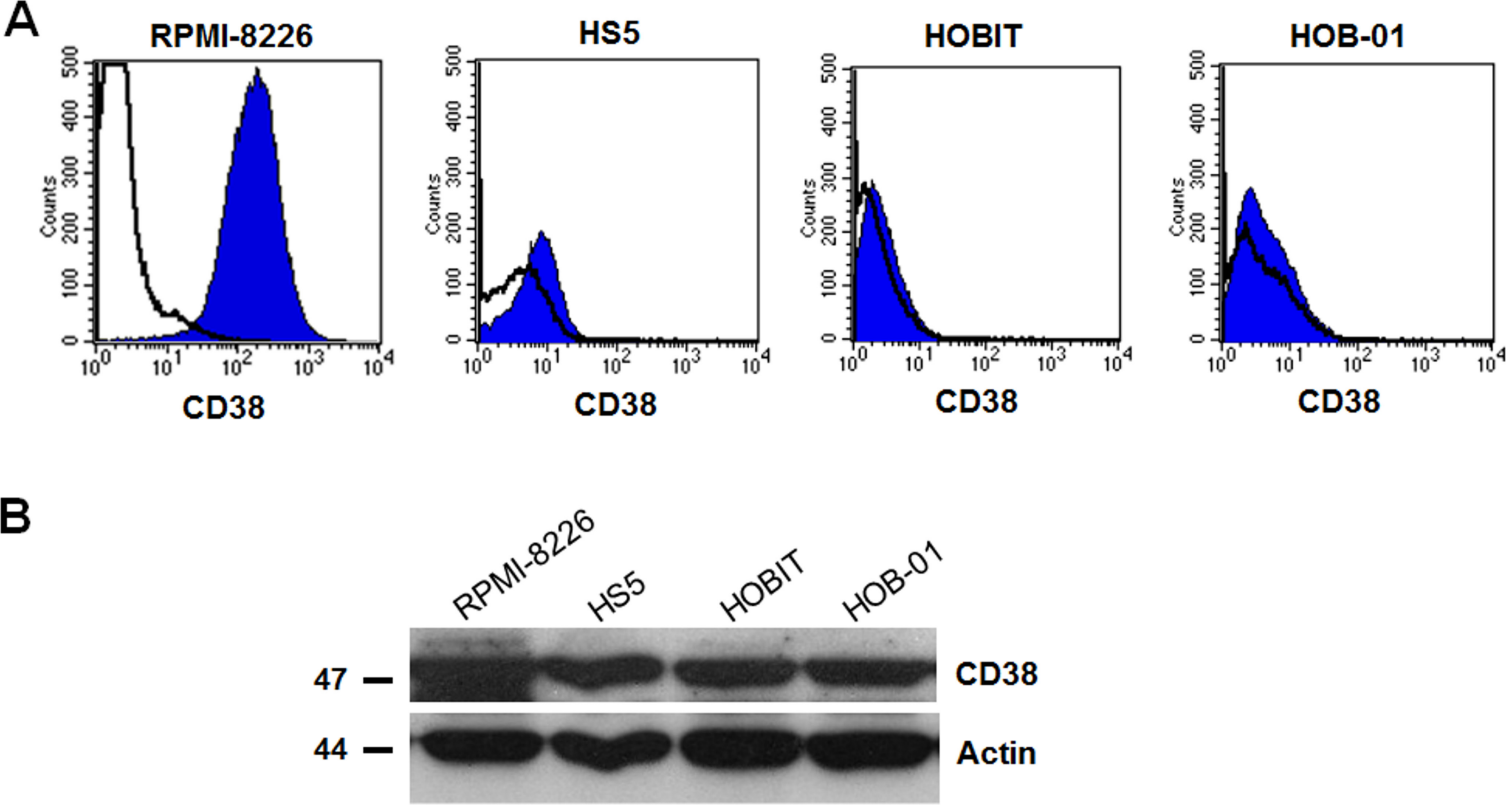
CD38 was present in the cytoplasm but it was not expressed at the surface by stromal and osteoblastic cells (**A**) CD38 expression was evaluated on HMCL RPMI-8226, on human stromal cell line (HS5), on immortalized human osteoblast-like cells (HOBIT) and on human pre-osteocytic cell line (HOB-01) by flow-cytometry. (**B**) CD38 citoplasmatic expression was evaluated on the same cells by western blot. HMCL RPMI-8226 was used as positive control and β-actin as internal control.

The expression pattern of CD38 was evaluated by flow cytometry on primary CD138+ purified from 16 MM patients, human myeloma cell lines (HMCLs) and microenvironment cell lines. All primary MM cells expressed CD38 at high level, as well as HMCLs (except for INA-6). The stromal cell line human stromal cell line (HS5) expressed CD38 weakly in contrast to human osteoblastic cell lines human pre-osteocytic cell line (HOB-01) and human osteoblast-like cells (HOBIT) which did not express CD38 at surface level (Figure 1A). On the other hand, CD38 expression was present at cytoplasmic level in the same cell lines (Figure 1B).

**Figure 2:**
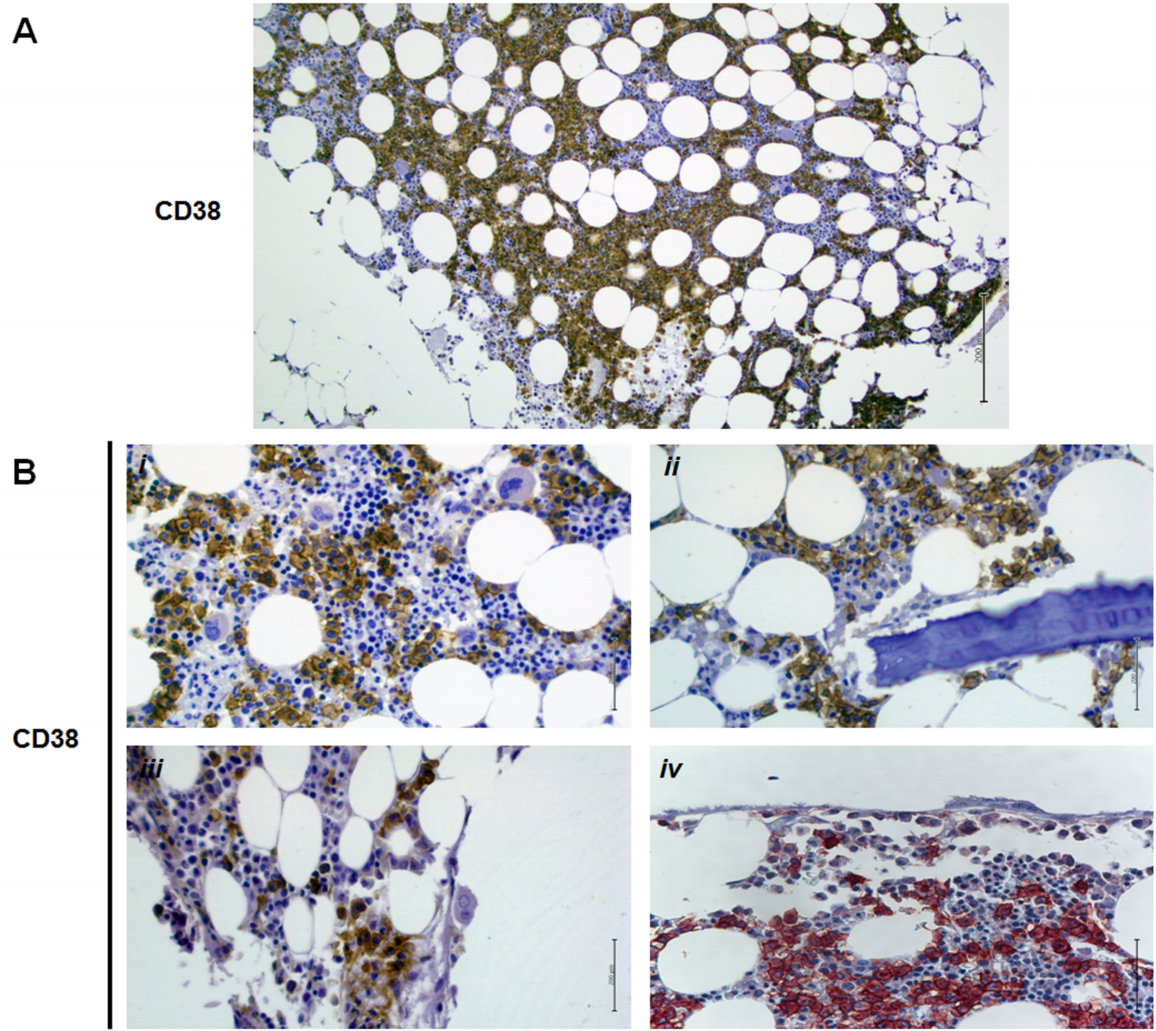
CD38 expression on bone biopsies of MM patients CD38 immunohistochemistry was performed on bone biopsies obtained from MM patients. Photos show representative MM patients. A strong CD38 immunostaining positivity was detected in PCs (**A**, **B i–iv**). OBs (B *ii*) and OCs (B *iii* and *iv*) were negative. The immunoperoxidase technique (*i–iii*) and Fast Red technique (*iv*) were used to reveal immunostaining. Original magnification 10× (A) and 40× (B).

The immunohistochemistry analysis performed on bone biopsies of MM patients confirmed the immunophenotype data. Specifically, MM cells expressed CD38 at high level (Figure 2A–2B), whereas OBs (Figure 2B, 2ii), OCs (Figure 2B, 2iii–iv) and endothelial cells did not. Moreover, the expression profiling of the nonsubstrate ligand CD31 and that of other ectoenzymes of the adenosinergic pathway, as CD39, CD73 and CD203a was also evaluated. Results are reported in Supplementary Tables 1, 2 and 3. Illustrative pictures of selected ectoenzymes distribution on bone biopsies of MM patients are showed in the Supplementary Figure 1.

### CD38 is expressed by monocytes and early OC progenitors but lost during the *in vitro* osteoclastogenesis

**Figure 3:**
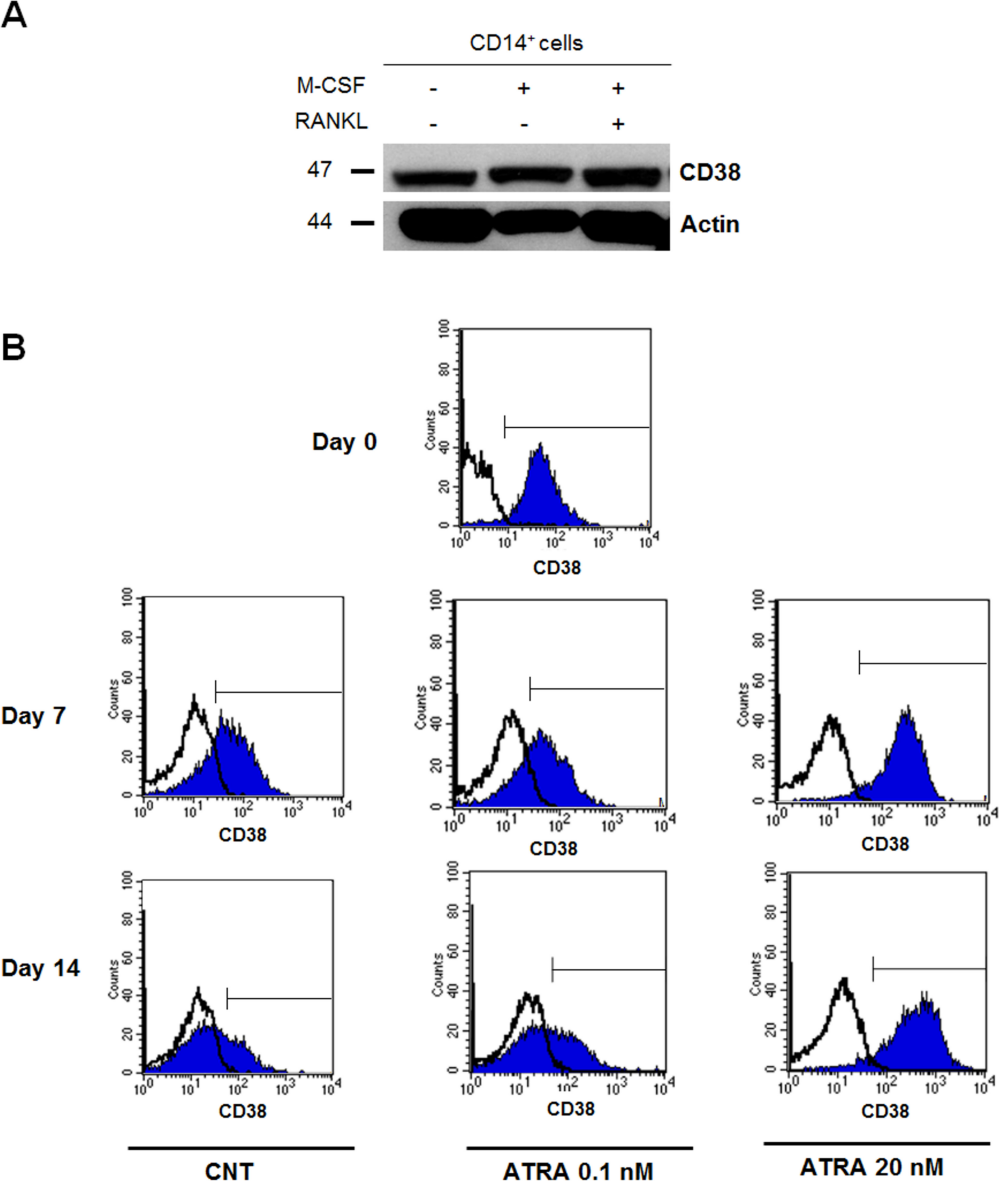
CD38 expression during *in vitro* osteoclastogenesis (**A**) CD38 expression was evaluated on CD14^+^ cells purified from PBMCs of HDs by an immunomagnetic method and seeded (1 × 10^6^ cells) in Petri dishes in αMEM at 10% FBS with rhM-CSF 25 ng/ml, in presence or absence rhRANKL 60 ng/ml. After 21 days of culture, cells were collected and analyzed by Western Blot. β-actin was used as internal control. (**B**) A time-course analysis of CD38 expression was performed during OC differentiation. CD38 expression was analyzed by flow-cytometry on CD14^+^ cells at seeding (day 0); cells (1 × 10^6^/ml) were cultured in 6-weel plates in αMEM at 10% FBS with rhM-CSF 25 ng/ml and rhRANKL 60 ng/ml, in presence or absence of ATRA (0.1 and 20 nM). On day 7 and 14 of culture period, cells were collected and CD38 expression was evaluated by flow-cytometry. (Graph represent flow-cytometry data from a representative experiment. Open histograms: negative control, blue histograms: anti-CD38).

We evaluated CD38 expression by monocytes and during *in vitro* osteoclastogenesis from CD14^+^ cells, either at cytoplasmatic levels by western blotting or surface levels by flow-cytometry. We found that both monocytes and mature OCs expressed CD38 at cytoplasmatic level (Figure 3A). Conversely, the flow-cytometry analysis showed that monocytes were positive for CD38 and that its expression decreased on day 7, with a further reduction on day 14 of OC differentiation (Figure 3B, left panels) suggesting that mature OCs lost CD38 surface expression. Interestingly, CD38 levels increased during *in vitro* osteoclastogenesis in the presence of high concentration (20 nM) of ATRA but not at low concentration (0.1 nM) (Figure 3B).

**Figure 4:**
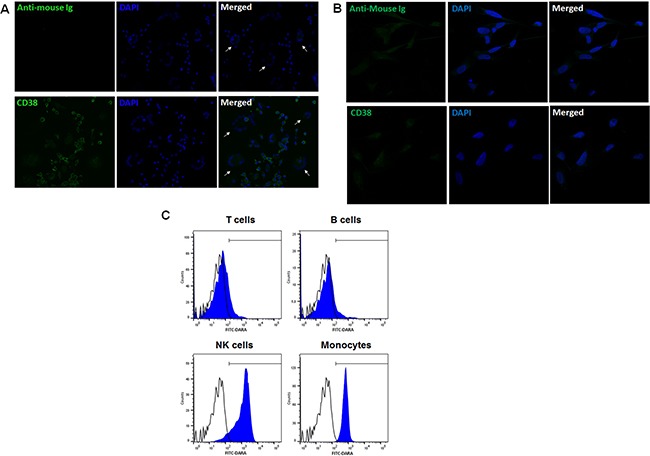
Anti-CD38 monoclonal antibodies bind monocytes but not mature OCs (**A**) OCs were differentiated from HD CD14^+^ cells, seeded on cover glasses, in 24-well plates, in αMEM with 10% FBS, rhM-CSF 25 ng/ml and rhRANKL 60 ng/ml, for 21 days. (**B**) HOBIT cell line was seeded on cover glasses until the confluence in DMEM 10% FBS. At the end of culture period, cells were stained with the primary antibody anti-CD38 IB4 followed by Alexa fluor-488-conjugated secondary antibody (lower images). The negative control samples were not incubated with primary antibody and are shown in the upper images. OCs were identified as multinucleated cells (≥ 3 nuclei, DAPI stained after permeabilization) and indicated by arrows. (**C**) PBMCs isolated from 5 HDs were incubated with FITC-conjugated DARA for 30 min at 4°C and then stained for the expression of surface markers CD3, CD19, CD16 or CD14 to identify T cells, B cells, NK cells and monocytes, respectively. Flow cytometry data from a representative experiment are reported in the graph. (Open histograms: negative control; blue histograms: FITC-DARA).

Confocal analysis confirmed that mature OCs did not express CD38 on their surface (Figure 4A). Lastly, we confirmed that OBs were negative for surface CD38 (Figure 4B).

### DARA inhibited OC formation, targeting early OC progenitors

Considering the CD38 expression profiling, we checked the ability of DARA to bind monocytes compared to other peripheral blood (PB) cell populations. We performed a flow cytometry assay with FITC-conjugated DARA on Healthy donor (HD) PB mononuclear cells (PBMCs) and demonstrated that DARA binds monocytes and NK cells. However, DARA binding was not detectable on T cells and B cells (Figure 4C), consistent with the CD38 expression previously described on monocytes.

**Figure 5:**
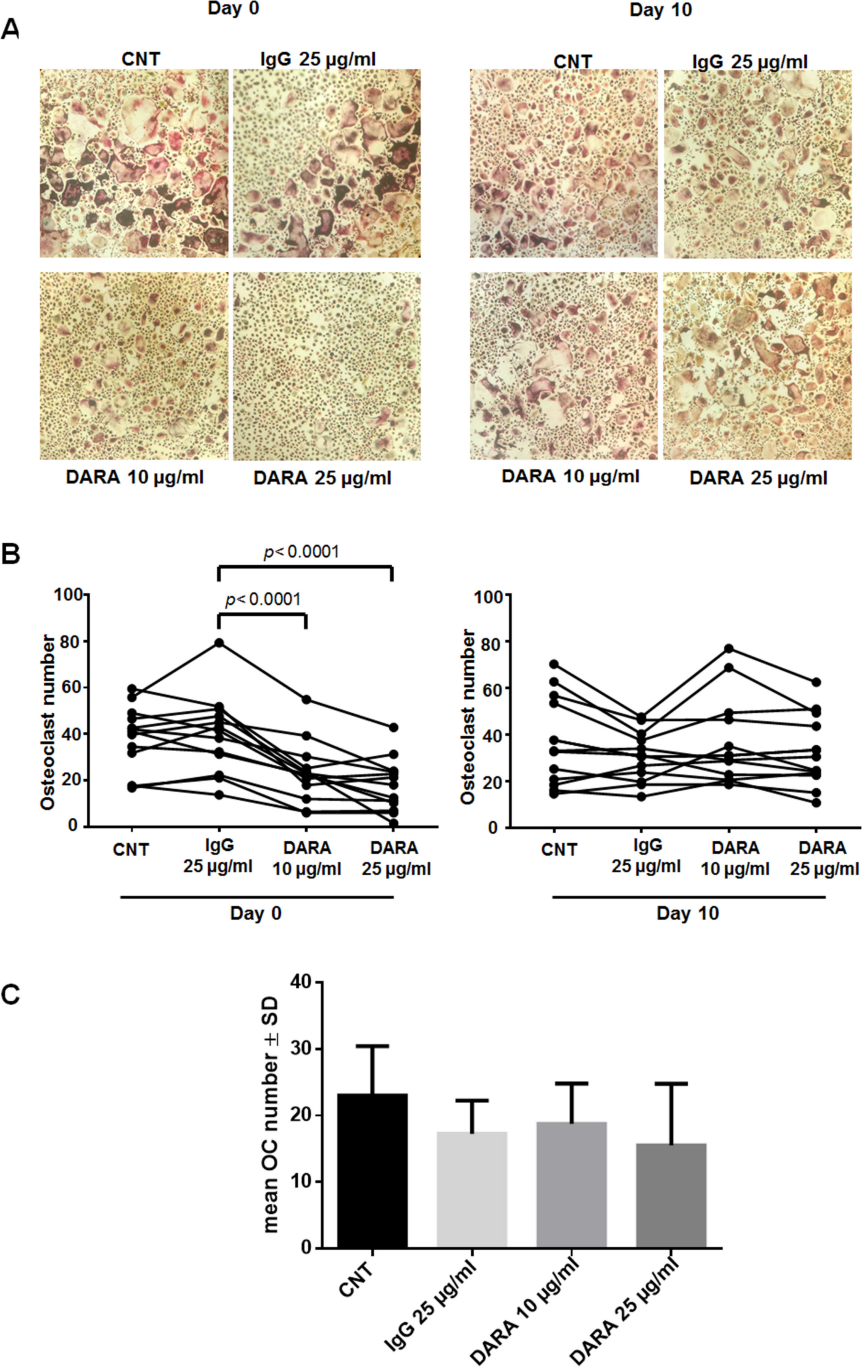
DARA inhibits *in vitro* osteoclastogenesis from MNCs but not from CD14^+^ cells affecting early OC progenitors (**A**, **B**) OCs were differentiated from MNCs of 13 MM patients, in the presence of DARA or isotype control IgG and then cultured for 21 days. DARA treatment was performed either at seeding (day 0) or after 10 days of culture (day 10). The OCs were identified and counted by light microscopy at the end of the culture period as multinucleated (≥ 3 nuclei) cells, positive for tartrate resistant acid phosphatase (TRAP) assay. Graphs represent the mean OC number of each individual patient (*p* calculated by two-tailed, paired Student's *t-test*) of 13 independent experiments. (**C**) Osteoclastogenesis was performed from purified CD14^+^ cells obtained from BM samples of MM patients cultured with rhM-CSF 25 ng/ml and rhRANKL 60 ng/ml for 21 days, in the presence of DARA (10–25 μg/ml) or isotype control IgG. The OCs were identified and counted by light microscopy at the end of the culture period as multinucleated (≥ 3 nuclei) cells, positive for TRAP assay.

**Figure 6:**
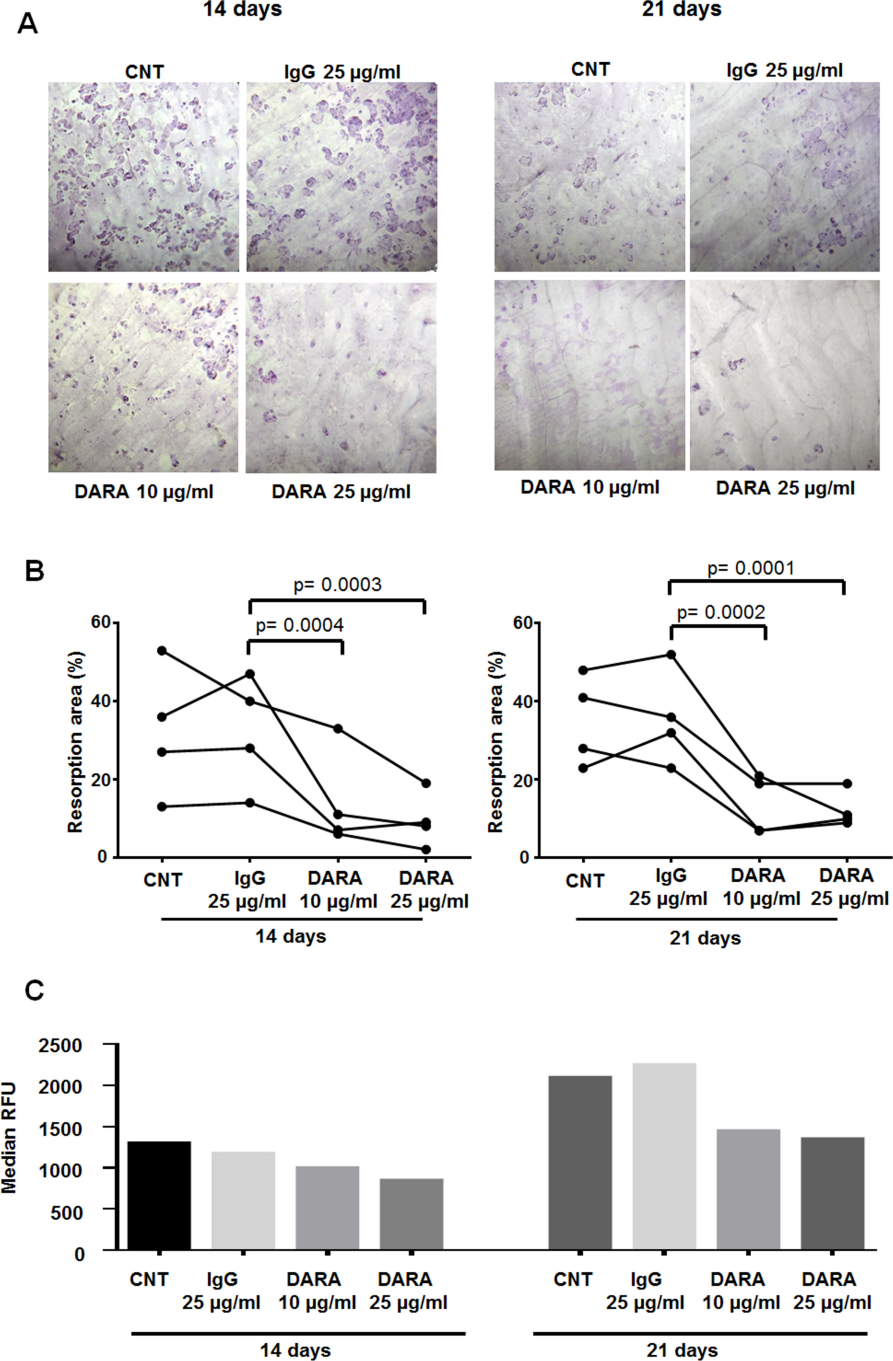
DARA decreases bone resorption area and OC activity (**A**, **B**) OCs were differentiated from MNCs of 8 MM patients in the presence of DARA or isotype control IgG and then cultured for 14 days (*n* = 4) and 21 days (*n* = 4) on bone slices. Pit area was observed under light microscope (10×). Data are expressed as the percentage of resorption area of 8 independent experiments (at least 3 replicated each condition). Symbols connected by a line represent cultures from the same donor. *p* calculated using a two-tailed, paired Student's *t-test*. (**C**) OC bone resorption activity was assessed by the OsteoLyse^™^ Assay. MM BM MNCs were seeded on a 96-well plate coated with fluorophore-derivatized human bone matrix and cultured for 14 or 21 days in αMEM with 10% FBS, rhM-CSF 25 ng/ml and rhRANKL 60 ng/ml in the presence of DARA (10–25 μg/ml) or isotype control IgG. The resorptive activity of the OCs was measured by sampling the cell culture supernatant at the end of differentiation, mixed with a Fluorophore-Releasing Reagent in a second 96-well assay plate and counted using time-resolved fluorescence by EnSpire Multimode Plate Reader 2300. Graph bars represent the median RFU on day 14 and day 21 of OC differentiation from a representative experiment.

Thereafter, to determine the potential effect of DARA on osteoclastogenesis, we performed *in vitro* OC differentiation from mononuclear cells (MNCs) of 13 MM patients, in the presence of DARA (or isotype control IgG) at seeding (day 0) or after 10 days of culture (day 10). When compared with cultures with IgG alone, the presence of DARA reduced the number of OCs when added on day 0 (two-tailed, paired Student's *t-test*, DARA 10 μg/ml vs IgG and DARA 25 μg/ml vs IgG: *p* < 0.0001) (Figure 5A–5B). On the contrary, DARA did not inhibit OCs formation when it was added on day 10, indicating the lack of effects on late OC progenitors and mature OCs (Figure 5A–5B). The inhibitory effect of DARA on OCs formation was not observed when OCs were differentiated from purified CD14^+^ cells (Figure 5C), consistent with DARA mechanisms of action in MM cells mediated by effector cells [22]. Treatment with DARA also decreased the area of osteoclast bone resorption pits and OC resorption activity (Figure 6) after both 14 (two-tailed, paired Student's *t-test*, DARA 10 μg/ml vs IgG: *p* = 0.0004; DARA 25 μg/ml vs IgG: *p* = 0.0003) and 21 days of treatment (two-tailed, paired Student's *t-test*, DARA 10 μg/ml vs IgG: *p* = 0.0002; DARA 25 μg/ml vs IgG: *p* = 0.0001).

In addition, we found that the inhibitory effect of DARA in OC differentiation was not mediated by soluble factors secreted by MM cells, since the conditioned medium (CM) of HMCLs previously pre-treated with DARA did not inhibited OC *in vitro* formation (data not shown). Lastly, we also investigated the potential effect of DARA-induced MVs on OC formation and we performed *in vitro* osteoclastogenesis from MM MNCs, in the presence of MVs isolated from the HMCLs RPMI-8226 and JJN3, treated with DARA 200 μg/ml or IgG. No significant differences in OC number were observed in the presence of DARA-induced MVs compared to IgG-induced MVs (Supplementary Figure 2).

### ATRA treatment increased DARA effects on OC formation

Since literature data showed that ATRA improves DARA-mediated ADCC and CDC against MM cells [23], we investigated the effect of DARA in combination with ATRA on *in vitro* osteoclastogenesis. Initial experiments were performed to evaluate the effect of ATRA (0.01 nM–200 nM) on OCs differentiated from MNCs of MM patients for 21 days. ATRA caused a dose-dependent inhibition of osteoclastogenesis with a minimal effect at 0.1 nM. Indeed, higher concentration induced a drastic reduction of osteoclasts (Supplementary Figure 3). In order to assess a possible combinatory effect, we used ATRA at 0.1 nM and 20 nM for further experiments.

**Figure 7:**
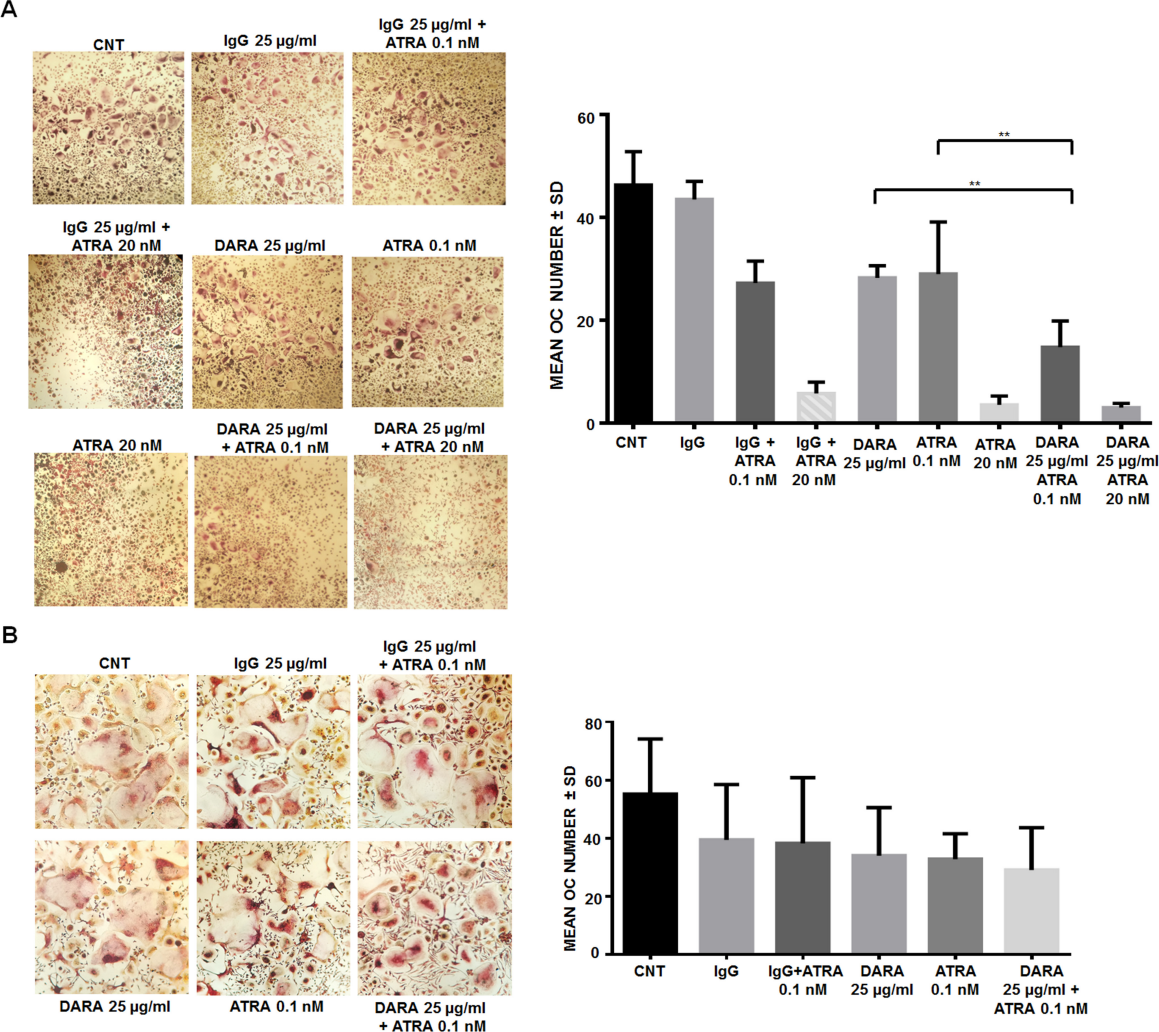
ATRA treatment increases DARA effects on OC differentiation (**A**) OCs were differentiated from MNCs of MM patients in the presence of DARA 25 μg/ml or isotype control IgG, with or without ATRA at 0.1 nM and 20 nM, or vehicle (DMSO), for 21 days. The OCs were identified and counted by light microscopy at the end of the culture period as multinucleated (≥ 3 nuclei) cells positive for TRAP assay. Graph bars represent the mean ± SD OC number for each well (*p* calculated by one-way ANOVA followed by Dunnett's multiple comparison test of a representative experiment **= *p* < 0.001). (**B**) Osteoclastogenesis was performed from purified CD14^+^ cells obtained from BM samples of MM patients cultured with rhM-CSF 25 ng/ml and rhRANKL 60 ng/ml for 21 days, in the presence of DARA (25 μg/ml) or isotype control IgG, with or without ATRA (0.1 nM), or vehicle (DMSO), for 21 days. The OCs were identified and counted by light microscopy at the end of the culture period as multinucleated (≥ 3 nuclei) cells, positive for TRAP assay.

OC number was significantly reduced in the combined treatment condition (DARA 25 μg/ml and ATRA 0.1 nM) compared to the single agent treatment (one-way ANOVA, DARA 25 μg/ml plus ATRA 0.1 nM vs DARA 25 μg/ml: *p* < 0.01; DARA 25 μg/ml plus ATRA 0.1 nM vs ATRA 0.1 nM: *p* < 0.01) (Figure 7A–7B). On the other hand, the treatment with ATRA at 20 nM showed a drastic effect on OCs which made the study of the effects in combination with DARA impossible. Yet, DARA 25 μg/ml plus ATRA 0.1 nM was used to treat purified CD14^+^ cells under osteoclastogenic conditions. The treatment did not affect the number of OC compared with the single agent treatment confirming the importance of effector cells in the system.

## DISCUSSION

The use of the CD38 molecule as a target for antibody-mediated treatment of MM [27] provided an opportunity to access evidence from *in vivo* applications. The anti-MM effect of DARA has been widely described. [19] Some issues, stemmed from the fact that the target molecule is expressed not only by the tumor but also by effectors and inhibitory cells, are still unresolved [28]. Several studies have reported the CD38 involvement in bone remodelling, in mice and rabbit models [16, 17], where both OBs and OCs expressed CD38. However, reports on CD38 expression by human OBs and OCs are still very limited [18, 29–31].

It has been reported that CD38 is also involved, in conjunction with other ectoenzymes such as CD73 and CD203a, in the alternative production of ADO. Horenstein AL. *et al*. [32] recently have shown that this ectoenzymatic network is active even in MM bone niche and that ADO levels correlate with disease aggressiveness and ISS staging of MM patients [32].

The study firstly focused on the evaluation of the expression profiling of CD38 on primary CD138 purified from MM patients. All MM samples expressed CD38 as well as HMCLs. We observed that human stromal and osteoblastic cell lines did not express CD38. However, OBs expressed CD38 at cytoplasmatic levels consistent with CD38 localization in endoplasmic reticulum and nuclear membrane, as found in osteoblastic cell lines by Sun L *et al*. [18]. Moreover, a study from Romanello M *et al*. [29] showed that OBs display ADP-ribosyl cyclase/CD38 activity and NAD+ stimulation inhibited cell growth, markedly altered cell morphology, and induced significant increases in alkaline phosphatase activity and osteocalcin mRNA, indicating that the nucleotide may act as a differentiation signal [29]. The analysis of gene expression of *CD38* by MM PCs did not show any correlation with the presence of osteolytic lesions in MM patients (Toscani D et al unpublished data).

In line with the literature [6, 7], the immunohistochemistry data showed that CD38 was expressed at high levels by PCs of patients with monoclonal gammopathies. On the other hand, OBs and OCs were negative for CD38 expression. MM cells also expressed CD31, CD39 and CD73 at variable levels, as previously reporte [10, 33, 34]. Interestingly, we found that OBs were positive for CD73 but expressed low levels of CD39 and did not express CD31. Previous studies showed that osteoblastic differentiation of hMSCs was induced by ATP degradation mediated by CD73 and CD39 expression on hMSC membrane [35], and that osteoblastogenesis is characterized by a stability of surface CD73, an increased CD203a expression and a lack of cell surface expression of CD38 and CD157 [36]. Lastly, endothelial cells express high level of CD31 and CD39, CD73 at variable levels while express neither CD38 nor CD203a. The distribution of the analyzed ectoenzymes within the MM microenvironment could clarify the involvement of bone cells in the ADO-mediated stimulation of growth and survival of MM cells.

Thereafter, we investigated the possible role of CD38 in human OC differentiation by the analysis of CD38 expression on monocytes and OC. Both cell types expressed CD38 in the cytoplasm in line with literature data [30, 31]. Faust J *et al*. [30] found that CD38 was expressed by OC-like cells, whereas a more recent study reported that mature OCs express CD38 [31]. Moreover, we checked flow cytometry expression of CD38 during *in vitro* osteoclastogenesis from CD14^+^ cells. We found that monocytes expressed CD38 at seeding but it was lost during OC differentiation. Confocal microscopy confirmed this evidence. This is in line with what Pfister M *et al*. [37] reported on macrophage differentiation. Overall these results indicate that, other than in PCs, the cell surface CD38 expression is limited to early osteoclast progenitors. Consequently, these cell types could be the target of anti-CD38 therapeutic Abs.

DARA, a human anti-CD38 IgG1κ antibody, has shown encouraging results in the treatment of MM [19, 22, 38, 39]. The main mechanisms by which DARA exerts its anti-myeloma effect are ADCC and CDC [22, 38]. However, DARA effects on bone cells and on MM-induced bone remodelling alterations are still unknown. Thus, based on CD38 expression profiling, we evaluated the effect of DARA on osteoclastogenesis. The flow-cytometry analysis showed that DARA binds monocytes and our *in vitro* experiments indicated that DARA treatment significantly reduced the number of TRAP-positive multinucleated OCs compared to IgG isotype controls when it was added on day 0 but not on day 10, consistent with the expression profiling of CD38 observed by flow cytometry. Furthermore, DARA significantly reduced the area of osteoclast bone resorption pits and OC activity. Interestingly, we showed that DARA inhibited OC formation only when OCs were differentiated from total MNCs but not from isolated CD14^+^ cells, suggesting that DARA effect on osteoclastogenesis was mediated by the effector cells, as shown for the anti-MM activity [22]. In support of our findings, a recent study from An G *et al*. [31] showed that a different humanized Ab specific for CD38, namely SAR 650984, had no effect on osteoclastogenesis from isolated CD14^+^ cells [31].

More recently, Horenstein AL *et al*. [26] have shown that DARA ligation on MM cells was followed by aggregation, polarization, and release of MVs, which express several molecules clustered in lipid domains on their surface, including both CD38 and DARA. However, the fate of MVs is still unknown. Chillemi A *et al*. [40] also showed a tendency of DARA-labeled MVs to cluster around NK cells and monocytes, since DARA shows a high affinity to Fc receptors of immune cell types [40]. Relying on these data, we investigated the involvement of DARA-induced MVs from MM cells in OC formation. We did not find any appreciable effects on *in vitro* osteoclastogenesis in the presence of DARA-induced MVs compared to IgG-induced MVs. Overall, our *in vitro* evidence indicates that the anti osteoclastogenic effect of DARA is mainly due to its ability to bind monocytes and OC progenitors, and, consequently, to the activation of effector cells. Since literature data showed that ATRA treatment increased DARA anti-MM effects, [23] we also tested the effect of DARA in combination with ATRA in osteoclastogenesis. High concentration of ATRA (20nM) enhanced CD38 expression on day 7 and 14 of OC differentiation, in line with that reported by Drach J *et al*. [41] on myeloid cells. On the other hand, low concentration of ATRA (0.1 nM) had no effect on CD38 expression on OC progenitors, but it decreased the number of TRAP positive multinucleate OCs when used in combination with DARA, compared with the single agent treatment supporting the possibility to use ATRA to enhance DARA effect. This combinatory effect is not likely to be correlated to the modulation of CD38 expression on OC progenitors by ATRA that occurs only at high concentration. As reported in the literature, [42] a direct inhibitory effect of ATRA on RANKL-stimulated osteoclastogenesis was shown and this mechanism could potentiate the effect of DARA on OC formation. Indeed, the combination ATRA plus DARA did not affect the number of OCs differentiated from purified CD14^+^ cells thus confirming the importance of effector cells and elucidating the effect of the combination. In conclusion, our findings define the expression profiling of CD38 and a panel of selected ectoenzymes and molecules in the bone niche of MM patients. Moreover, consistent with the CD38 expression profiling, we show that therapeutic anti-CD38 DARA significantly inhibits OC formation by targeting OC progenitors with a potential clinical relevance. On the other hand, CD38 was expressed by OBs at detectable levels, which *bona* *fide* rules out the possibility of cytotoxic events delivered by DARA. Taken together, these findings highlight the possibility of a role of CD38 during OC formation, which further supports the use of DARA as a treatment for bone destruction in MM patients.

## MATERIALS AND METHODS

### Patients

BM aspirates were obtained from 16 MM consecutive patients, including 8 newly diagnosed (ND) (median age: 70 years, range: 57–86; 75% Female (F), 25% Male (M); International Staging System (ISS): I = 12.5%, II = 12.5%, III = 75%) and 8 relapsed (R) (median age: 76 years, range: 43–90; 38% F, 62% M; ISS: I = 12.5%, II = 25%, III = 62.5%), to purify fresh CD138^+^ cells and evaluate the immunophenotypic profiling. Only samples with purity > of 90% checked by flow cytometry (BD FACS Canto II with Diva software; Becton, Dickinson and Company (BD); Franklin Lakes, NJ) were tested.

A retrospective study of a total cohort of 51 patients with monoclonal gammopathies was performed including 25 patients with ND MM: median age 70 years range: 41–85; 36% F, 64% M; ISS: I = 30%, II = 22%, III = 48%; 12 with smoldering MM (SMM): median age: 70 years, range: 47–83 years, 33% F, 67% M; and 14 with monoclonal gammopathy of uncertain significance (MGUS): median age: 71 years, range: 39–88 years; 64% F 36% M; who had access to Hematology Unit of Parma from May 2011 to June 2015. Bone biopsies were obtained from iliac crest of each patients to perform immunohistochemistry for the following antigen: CD38, CD39, CD73, CD31, and CD203a.

Patient samples were obtained after informed consent, according to the Declaration of Helsinki. The study was included in a larger project on patients with monoclonal gammopathies, approved by the Institutional Ethical Review Board of our Hospital.

### Drugs

DARA and isotype control IgG were provided by Janssen Pharmaceuticals (Spring House, PA, USA). ATRA was purchased from Sigma- Aldrich (Saint Louis, MO).

### Cells and cell culture conditions

#### PC purification

BM CD138^+^ cells were purified from total MNCs by an immuno-magnetic method using anti-CD138 mAb coated microbeads (MACS, Miltenyi Biotec; Bergisch-Gladbach, Germany), as previously described [43].

#### DARA treatment on *in vitro* OC formation

OCs were obtained either from total BM MNCs of MM patients, or purified CD14^+^ cells from PBMCs of 3 HDs. Cells were seeded in αMEM with 10% FBS, rhM-CSF 25 ng/ml and rhRANKL 60 ng/ml (Peprotech; Rocky Hill, NJ) in the presence of DARA (10–25 μg/ml) or isotype control IgG added to culture medium on day 0 or after 10 days, with or without ATRA (0.1–20 nM) or vehicle (DMSO) for 21 days, replacing half medium every 2–3 days. BM MNCs were also incubated in the presence or absence of the CM (dilution ratio with *αMEM with 10% FBS, rhM-CSF 25 ng/ml and rhRANKL 60 ng/ml*, 1:3) of JJN3 and RPMI-8226 (5 × 10^5^ cells/ml) previously pre-treated with DARA (10-25 μg/ml) or isotype control IgG for 48 h, and cultured for 21 days replacing half medium every 2-3 days. Each condition was performed at least in triplicate. For dose-finding experiment, BM MNCs were cultured in *αMEM with 10% FBS, rhM-CSF 25 ng/ml and rhRANKL 60 ng/ml* (Peprotech; Rocky Hill, NJ) in presence of ATRA (0.01 nM–200 nM), or vehicle (DMSO) for 21 days, replacing half medium every 2–3 days.

MVs isolation, tartrate resistant acid phosphatase (TRAP) and Osteoclastogenesis resorption assays. Methods are detailed in the Supplementary Methods.

### Flow cytometry assay

The immunophenotype of primary BM CD138^+^ cells, HMCLs and microenvironment cells was analyzed with the following mAbs:

CD38-APC (clone HIT2, code n. 560677, BD)

CD31-FITC (clone WM59, code n. 555445, BD)

CD39-APC (clone eBioA1, code n. 17-0399-41, eBioscence; San Diego, CA)

CD73-APC (clone CB73), produced in the Lab of one of the authors (FM) and FITC-conjugated by AcZon (Bologna, Italy)

CD203a-FITC (clone 3E8, kindly provided by J. Goding)

CD14-PE (clone M5E2, code n. 555398, BD).

### DARA binding on PBMCs

PBMCs isolated from 5 HDs were washed in PBS containing 1% BSA + NaN_3_ and incubated with FITC-conjugated DARA for 30 min at 4°C. T cells, B cells, NK cells and monocytes were identified by expression of surface markers using the following mAbs produced in the Lab of one of the authors (FM) and conjugated by AcZon (Bologna, Italy), respectively: anti-CD3-PerCP Cy5.5 (clone CBT3G), CD19-APC (clone CB19), CD16-APC (clone CB16) and CD14-APC (clone CB14). Cells were washed in PBS and acquired on FACS. Data were analyzed using FlowJo Software (TreeStar, Ashland, OR).

### Immunohistochemistry

Patient bone biopsies were fixed in formalin at 10%. Once fixed, the samples were embedded in paraffin, so as to allow the cut to the microtome thin sections (3–6 μm). Bone biopsy sections were incubated with polyclonal rabbit Abs against CD38 (1:1.500) (code n. HPA022132, Sigma, Saint Louis, MO) or CD39 (1:70) (code n. 14211-1-AP, Proteintech, Manchester, UK), CD73 (1:1.000), CD31 (1:50), for 60 min at room temperature. Antibodies anti-CD31, CD73 and CD203a were produced in the Lab of one of the authors (FM). The staining was visualized using the UltraVision LP Large Volume Detection System HRP polymer (Thermo Scientific, Erembodegem, Belgium) according to the manifacture's specifications. A sample was considered positive if the target antigen was detected at least in the 50% of cells. Images were captured by DP22 digital camera (Olympus; Hamburg, Germany) and analyzed with the OLYMPUS Stream software, adjusting tone and contrast to ensure the best image quality.

### Western blot analysis

These methodologies were detailed in Supplemental Methods section of Supplementary Data.

### Immunofluorescence confocal microscopy

Immunofluorescence analysis was carried out to quantify CD38 expression on mature OCs, obtained from HD CD14^+^ cells. Cells (1.2×10^6^/well) were cultured in 24-well plates on Ø 12mm cover glasses (Thermo Scientific), in αMEM with 10% FBS, rhM-CSF 25 ng/ml and rhRANKL 60 ng/ml, for 21 days, replacing half medium every 2–3 days. At the end of culture period, cells were reacted at 4°C with the primary mAb anti-CD38 (clone IB4, produced in the Lab of one of the authors (FM) for 1 h. After rinsing with PBS, goat anti-mouse IgG (H+L) conjugated with Alexa Fluor 488 secondary Ab (code n. 115-545-003, Jackson ImmunoResearch Laboratories, West Grove, PA) was added for 30 min at 4°C. The negative control samples were not incubated with primary antibody. After extensive washings, the cells were fixed with paraformaldehyde (2% in PBS, 15 min at 4°C). DNA was visualized after permeabilization with 0.2% Triton X-100 detergent (5 min at 4°C) and successive staining with DAPI. The same protocol was used to quantify CD38 expression on HOBIT cell line. The confocal imaging was performed on a TCS SP5 laser scanning confocal microscope with 4 lasers (Leica Microsystems; Wetzlar, Germany).

### Statistical analysis

Data were expressed as means ± standard deviation (SD) or medians. Two-tailed, paired Student's *t-test* or one-way ANOVA followed by Dunnett's multiple comparison test were used to test significant differences. In all cases, *p* < 0.05 was considered significant. GraphPad Prism 6.1^™^ (GraphPad Software Inc., La Jolla, CA, USA) was used for all the statistical analyses.

## SUPPLEMENTARY MATERIALS FIGURES AND TABLES



## Abbreviations

MM: Multiple myeloma
PC: Plasma cell
OCs: Osteoclasts
OBs: Osteoblasts
BM: Bone marrow
ADO: Adenosine
DARA: Daratumumab
mAb: Monoclonal antibody
CDC: Complement-dependent citotoxicity
ADCC: Antibody-dependent cell-mediated citotoxicity
ADCP: Antibody-dependent cellular phagocytosis
ATRA: all-*trans* retinoic acid
MVs: Microvesicles
ND: Newly diagnosed
F: Female
M: Male
ISS: International staging system
SMM: Smoldering MM
MGUS: Monoclonal gammopathy of uncertain significance
MNCs: Mononuclear cells
PBMC: Peripheral blood mononuclear cell
HDs: Healthy donors
CM: Conditioned media
SD: Standard deviation
TRAP: Tartrate resistant acid phosphatase
HMCLs: Human myeloma cell lines
RFU: Relative fluorescence unit.
