# Supplementary material for: Expression of CD38 in myeloma bone niche: A rational basis for the use of anti-CD38 immunotherapy to inhibit osteoclast formation
Source: Oncotarget. 2017 May 16;8(34):56598–611. doi: 10.18632/oncotarget.17896 (PMC5593586; doi:10.18632/oncotarget.17896)
Supplement: Supplementary file 1 [file oncotarget-08-56598-s001.pdf]

Expression of CD38 in myeloma bone niche: A rational basis for the use of anti-CD38 immunotherapy to inhibit osteoclast formation

Supplementary Materials

MATERIALS AND METHODS

Cells and cell culture conditions

Cell lines

Human myeloma cell lines (HMCLs) JJN3, OPM2 and RPMI-8226 were purchased from DSMZ (Braunschweig, Germany). The HMCL U266 was obtained from the American Type Culture Collection (ATCC) (Rockville, MD), the HMCL XG1 was generously gifted by Martine Amiot (Nantes, France) and the HMCL INA-6 by Francesco Piazza (Padova, Italy). HMCLs were maintained in culture as previously described.26 The human pre-osteocytic cells HOB-01 were established from human bone and kindly provided by Julia Billars (Collegeville, PA). Immortalized human osteoblast-like HOBIT cells were kindly provided from Dr. B. L. Riggs (Rochester, MN). The human bone marrow stromal cell line HS-5 was purchased from ATCC. All cell lines were authenticated and tested for mycoplasma.

Microvesicles (MVs) isolation for OC formation

The HMCLs RPMI-8226 and JJN3 was seeded in 6-well plates at 1×10^6 cells/ml, in RPMI-1640 with 10% FBS, in presence or absence of DARA (200 µg/ml) or isotype control IgG for 1 h at 4°C to obtain MM derived MVs. After incubation, cells were collected, washed with PBS and then resuspended at 1×10^6 cells/ml in RPMI with 10% FBS depleted from MVs (obtained from serial ultracentrifugation) and incubated at 37°C for 24 h. Then, the supernatants were serially centrifuged at 700 × g and 3,000 × g for 10 min, and again at 15,000 × g for 90 min. Then, the supernatants were discarded and pellets were resuspended in *αMEM with 10% of FBS* depleted from MVs, supplemented with rhM-CSF 25 ng/ml and rhRANKL 60 ng/ml in order to obtain MV suspensions. BM MNCs from 6 MM patients were seeded in 96-well plates and cultured with the MV suspensions for 21 days. Half medium was replaced every 3 days with freshly isolated MV suspensions.

TRAP assay and osteoclastogenesis resorption assay

The OCs were identified and counted by light microscopy at the end of the culture period as multinucleated (≥ 3 nuclei) cells positive for tartrate resistant acid phosphatase (TRAP) assay (Sigma Aldrich).

Bone resorption ability of OCs was assessed by the OsteoLyse™ Assay using 96-well plate coated with fluorophore-derivatized human bone matrix (europium-conjugated collagen) and (Human Collagen) (Lonza; Walkersville, MD). The resorptive activity of the OCs was measured by sampling the cell culture supernatant at the end of cell culture. Briefly, 1 µL of the cell culture supernatants was added to 200 µL of Fluorophore-Releasing Reagent in a second 96-well assay plate and counted using time-resolved fluorescence by EnSpire Multimode Plate Reader 2300 (Perkin Elmer; Waltham, MA). The relative fluorescence unit (RFU) value obtained is directly proportional to OC number and the degree of OC differentiation.

Pit formation assay

Cells were handled like described above but seeded on bone slices from the beginning of the culture. Cells were cultured for 14–24 days under osteoclastogenic medium in the presence or absence of DARA (10–25 µg/ml) or isotype control IgG. At culture termination, the bone slices were transferred to 24-well plate and stained with acid hematoxylin. Then, cells were gently scraped off and resorption pits were visualized by light microscopy. At least three view fields were randomly selected for each bone slice for further pit area analysis which were quantified using a small point grid. The results were presented as the percentage of resorption area of the whole slice surface.

Western blot

Immunoblotting on monocytes and OCs was performed as previously reported[25] using the following antibodies: mouse monoclonal anti-CD38 (1:1,000)

(clone 4G3, code n. WH0000952M2, Sigma Aldrich) and mouse anti-β-actin monoclonal antibody (1:5,000) (clone AC-15, code n. A5441, Sigma-Aldrich) as internal control.

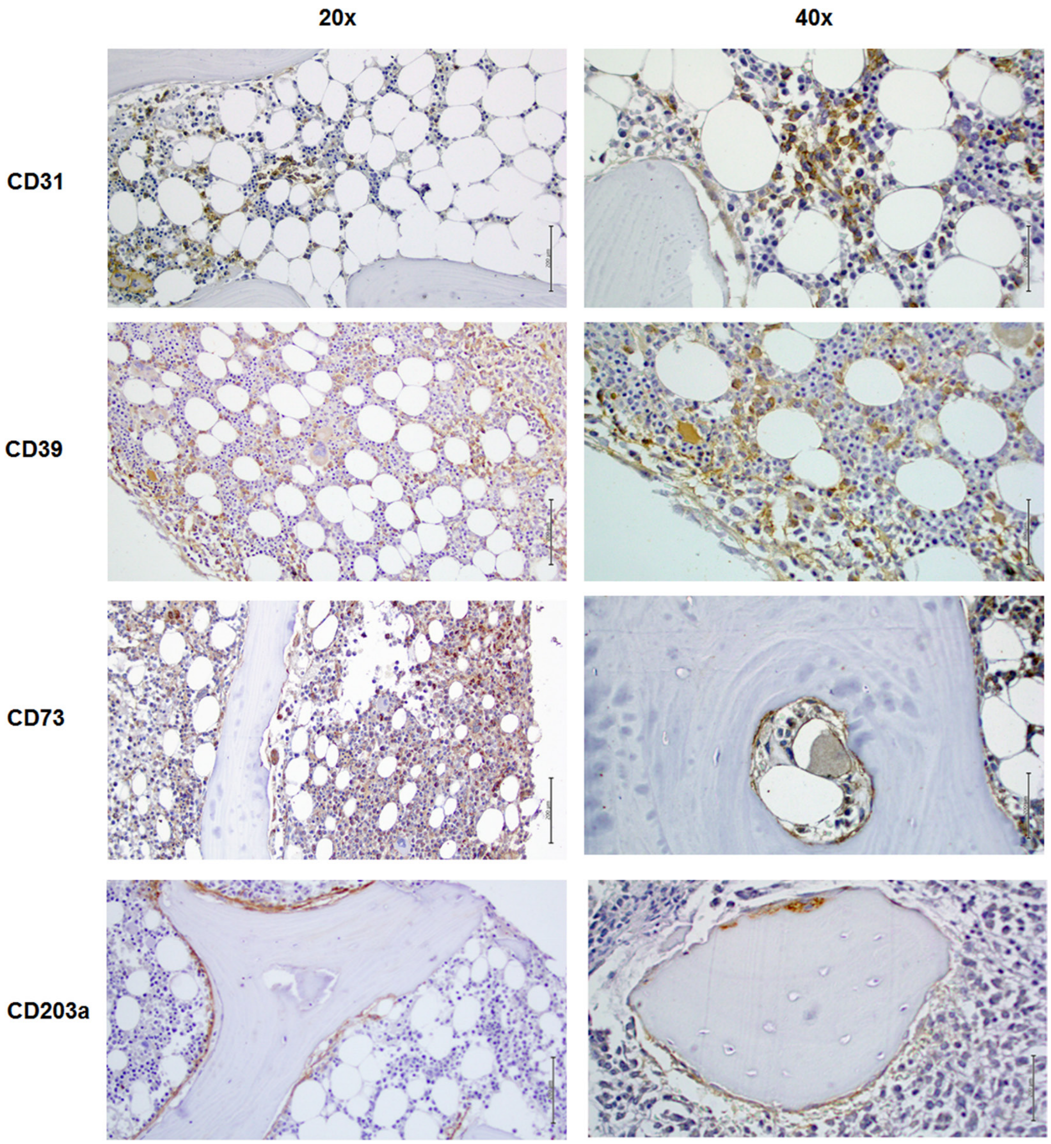

Supplementary Figure 1: CD38-selected ectoenzyme distribution on bone biopsies of MM patients. CD31, CD39, CD73 and CD203a immunostaining was performed on BM biopsies from MM patients and representative pictures are reported. The immunoperoxidase technique was used to reveal immunostaining. Original magnification 20× (left column), 40× (right column).

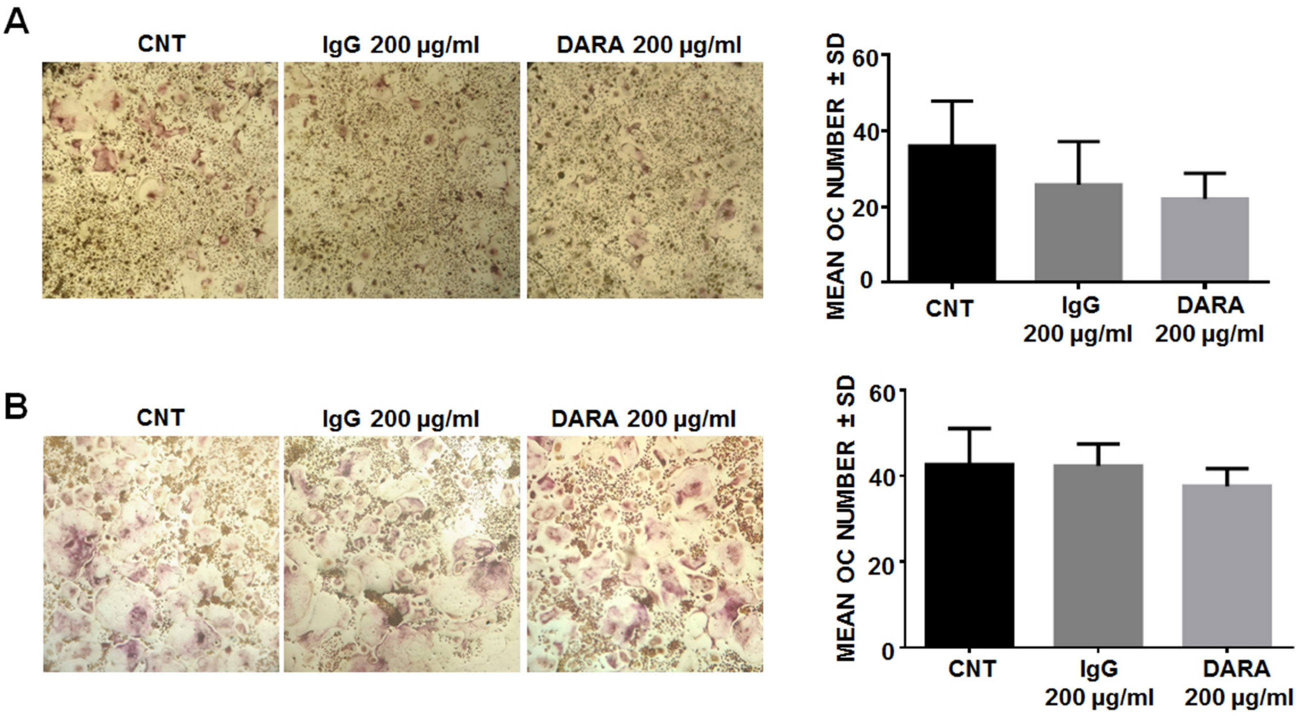

Supplementary Figure 2: DARA effect on OC formation was not significantly mediated by MV release. OCs were differentiated from MM BM MNCs in the presence of (**A**) RPMI-8226 or (**B**) JJN3-derived MVs. The HMCL RPMI-8226 was seeded in 6well plates at 1×10^6 cells/ml, in RPMI-1640 with 10% FBS, in presence or absence of DARA (200 µg/ml) or isotype control IgG for 1 h at 4°C. After incubation, cells were collected, washed with PBS and then resuspended at 1×10^6 cells/ml in RPMI-1640 with 10% FBS depleted from MVs and incubated at 37°C for 24 h. Then, the supernatants were serially centrifuged and finally MV pellets were resuspended in αMEM with 10% FBS depleted from MVs, rhM-CSF 25 ng/ml and rhRANKL 60 ng/ml in order to obtain MV suspensions. BM MNCs from MM patients were seeded in 96-well plates and cultured with the MV suspensions for 21 days. Half medium was replaced every 3 days with freshly isolated MV suspensions. The OCs were identified and counted by light microscopy at the end of the culture period as multinucleated (≥ 3 nuclei) cells positive for TRAP assay. Graph bars represent the mean OC number ± SD for each well of 4 (for A) and 2 (for B) independent experiments.

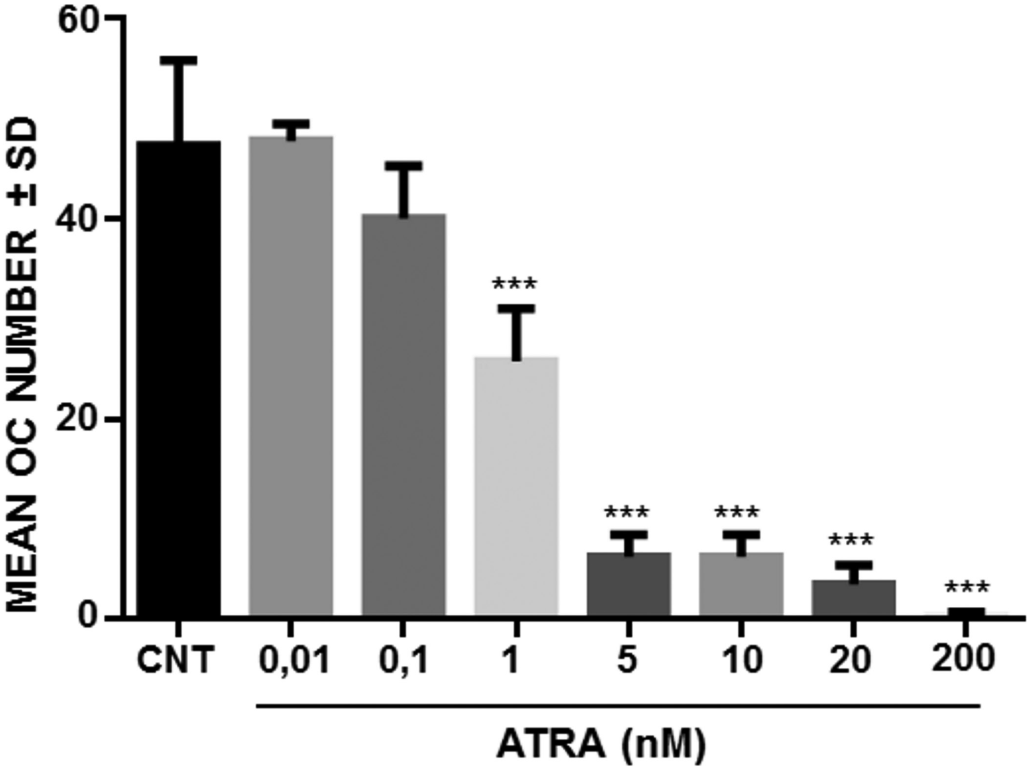

Supplementary Figure 3: ATRA effect on OC formation. OCs were differentiated from MM BM MNCs in the presence of different concentration of ATRA, ranging from 0.01 nM to 200 nM, or vehicle (DMO) for 21 days. The OCs were identified and counted by light microscopy at the end of the culture period as multinucleated (≥ 3 nuclei) cells positive for TRAP assay. Graph bars represent the mean OC number ± SD for each well of 1 independent experiment (***= $p < 0.001$).

Supplementary Table 1: Flow-cytometry analysis of CD31, CD39, CD73 and CD203a expression by CD138+ cells

Patients	CD31	CD39	CD73	CD203a
MM1	−	−	−	−
MM2	−	−	−	+
MM3	ND	+	−	+
MM4	+	+	−	+
MM5	ND	−	+	−
MM6	+	−	+	−
MM7	−	+	−	+
MM8	+	+	−	+
MM9	+	+	−	+
MM10	−	−	−	−
MM11	+	−	−	+
MM12	+	+	−	−
MM13	+	−	−	+
MM14	+	−	−	+
MM15	+	+	−	−
MM16	+	+	−	+

Abbreviations: MM, Multiple Myeloma; ND, Not Determined.

Supplementary Table 2: Flow−cytometry analysis of CD31, CD39, CD73 and CD203a expression by HMCLs and microenvironment cell lines

HMCLs				
	CD31	CD39	CD73	CD203a
JJN3	−	+	−	+
KMS-12-BM	−	+	−	+
XG1	+	+	−	+
RPMI-8226	−	−	−	+
INA-6	+	+	−	+
OPM2	−	−	−	+
**Microenvironment cells**				
	CD31	CD39	CD73	CD203a
HS-5	ND	−	+	+
HOB-01	ND	−	+	+
HOBIT	−	−	+	+

Abbreviations: HMCLs, Human myeloma cell lines; ND: Not Determined.

Supplementary Table 3: % of patients positive for CD31, CD39, CD73 and CD203a after immunostaining of bone biopsies

Plasmacells				
**Diagnosis**	CD31	CD39	CD73	CD203a
MGUS	80%	36%	11%	NEG
SMM	80%	58%	45%	NEG
MM	78%	39%	50%	NEG
**Osteoblasts**				
**Diagnosis**	CD31	CD39	CD73	CD203a
MGUS	NEG	NEG	78%	60%
SMM	NEG	17%	91%	64%
MM	NEG	4%	100%	78%
**Endothelial cells**				
**Diagnosis**	CD31	CD39	CD73	CD203a
MGUS	90%	93%	56%	NEG
SMM	100%	92%	64%	NEG
MM	100%	96%	50%	NEG

Abbreviations: MGUS: monoclonal gammopathy of uncertain significance, SMM: Smoldering Multiple Myeloma; MM: Multiple Myeloma; NEG: negative.
